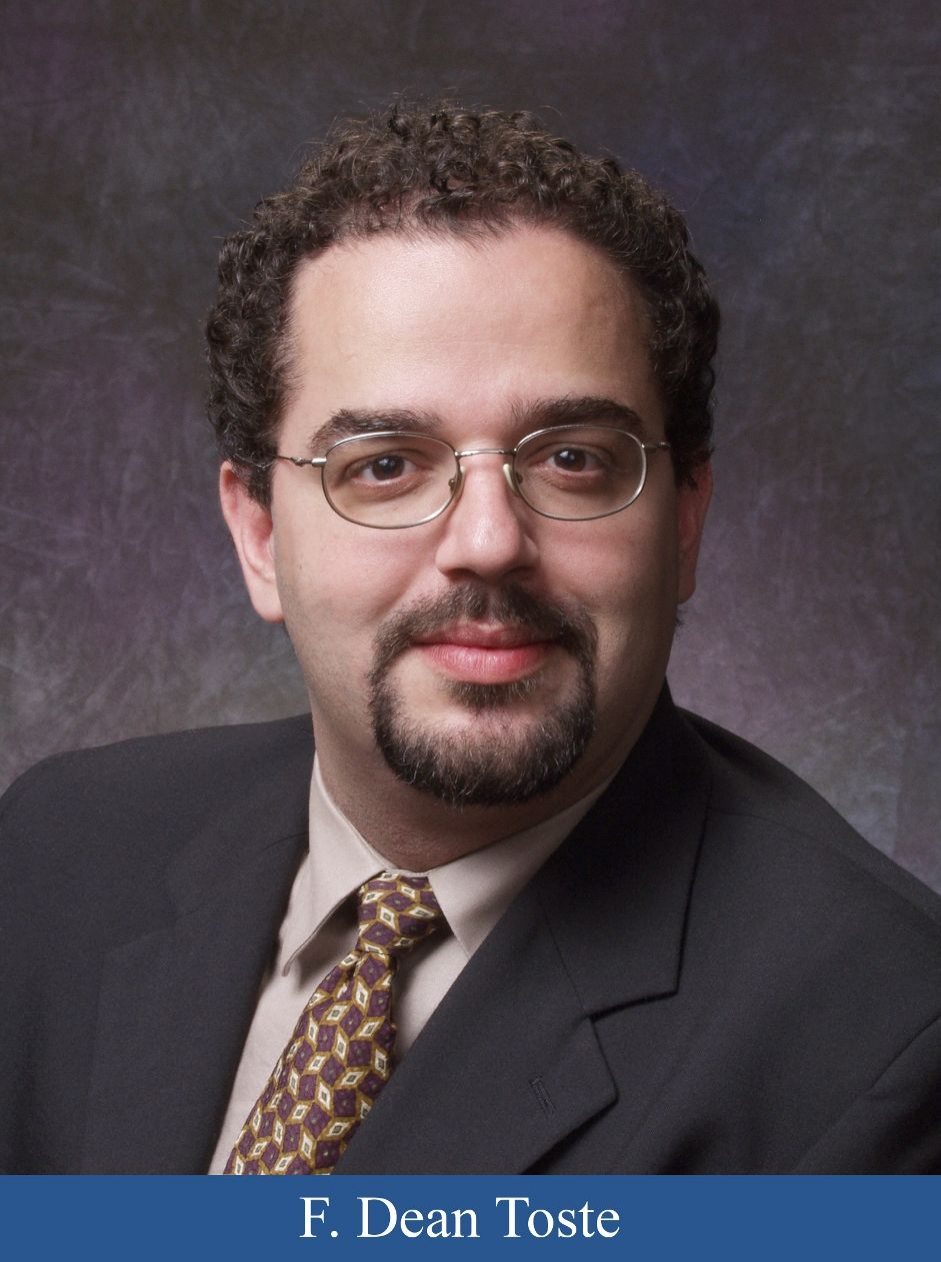# Gold catalysis for organic synthesis

**DOI:** 10.3762/bjoc.7.63

**Published:** 2011-05-04

**Authors:** F Dean Toste

**Affiliations:** 1 Department of Chemistry, University of California, Berkeley, CA, United States

The past decade has witnessed a remarkable growth in the number of organic reactions that are catalyzed by homogeneous gold complexes. Many of the early reported reactions took advantage of the propensity of gold complexes to serve as excellent catalysts for reactions that proceed through π-activation of carbon–carbon multiple bonds. Subsequently, the reactivity paradigms available for gold complexes have proven to be almost limitless. The contributions from over 25 research groups from 13 countries underscore the growing importance of homogenous gold catalysis. More importantly, the papers in this Thematic Series highlight the remarkable breath of reactivity that can be accessed using homogenous gold complexes as catalysts; from catalysis of sigmatropic rearrangement, cycloaddition and cycloisomerization reactions, to applications in enantioselective catalysis, oxidative coupling and the total synthesis of natural products, and transformations of alkynes, allenes, alkenes and even C–H bonds. A true treasure chest of reactivity!

I am grateful to all of the authors that have contributed to this Thematic Series. I hope you enjoy reading of their achievements as much as I have.

F. Dean Toste

Berkeley, April 2011